# A Qualitative Study of Tobacco Dependence Treatment in 19 US Dental Hygiene Programs

**DOI:** 10.5888/pcd9.120121

**Published:** 2012-11-01

**Authors:** Anne Koerber, Joan M. Davis, Nancy A. Newton

**Affiliations:** Author Affiliation: Joan M. Davis, Southern Illinois University, Carbondale, Illinois; Nancy A. Newton, Chicago School of Professional Psychology, Chicago, Illinois.

## Abstract

**Introduction:**

The US Public Health Service calls for health professionals to provide tobacco dependence counseling for patients. The purpose of this study was to understand how dental hygiene programs make decisions about and provide training for tobacco dependence counseling to help them graduate more fully competent hygienists.

**Methods:**

We conducted interviews (N = 32) with mainly program and clinic directors from 19 US dental hygiene education programs for this qualitative case study. We explored fluoride therapy training and tooth whitening training for comparison. Two analysts summarized the transcripts into a case study for each program.

**Results:**

All programs reported a similar process of learning about and choosing a method for teaching the topics explored. The programs used a common process, ADPIE (assess, diagnose, plan, implement, evaluate), to structure students’ clinical encounters. Almost all programs train students to ask about tobacco use and to advise quitting, but few programs train students to effectively help patients to quit and only 2 programs evaluated the competence of all students to provide such training. ADPIE shows promise for integrating tobacco dependence treatment more fully into the clinical training of dental hygiene students. Comparison to tooth whitening and fluoride therapy training indicated that complexity of the treatment and alignment with dental hygiene’s mission were themes related to training decisions.

**Conclusion:**

Full implementation of tobacco dependence counseling into dental hygiene education requires a commitment by dental hygiene educators to train students and faculty in counseling techniques and their evaluation. We identified an existing clinical structure as showing promise for facilitating improvement.

## Introduction

Tobacco use causes or complicates many chronic diseases, including diseases of the teeth, gums, and mouth such as caries, periodontal disease, and oral cancer (1). Although government public health planning commissions universally call for health care providers to provide tobacco dependence treatment (TDT) for patients, many dentists, dental hygienists, and other health care providers do not yet meet government recommendations. Although this gap has multiple facets, educational institutions may not be adequately training their graduates to provide TDT. 

National action plans such as Healthy People 2020, the 2003 National Action Plan for Tobacco Cessation, and the US Public Health Service (USPHS) recommend that all clinicians, specifically dental professionals, should consistently identify, document, and treat every tobacco user seen in a health care setting (2–4). The USPHS calls for clinicians to help patients who are not ready to quit to become ready and to help those who are ready with quitting strategies. Their evidence-based model is called “the 5 As”: 1) asking a patient about tobacco use, 2) advising a patient to quit tobacco use, 3) assessing the patient’s tobacco use and readiness to quit, 4) assisting the patient to quit, and 5) arranging for support and follow-up for the patient to quit. Their recommendations note that the efficacy of the tobacco dependence counseling is dose dependent; both frequency of interventions and time spent increase the efficacy of the counseling (4). Unfortunately, TDT including all 5As is rarely offered in dental settings (5–8) or other medical settings (7). Although many factors contribute to this, research demonstrates that training health professionals to use counseling techniques is an essential first step (9).

Our goal was to examine the processes and structures used by dental hygiene programs to train their students in TDT to inform the process of educating health professionals to help their patients quit using tobacco. We used qualitative methods to explore how dental hygiene education programs made decisions about incorporating tobacco dependence training in their programs: how they obtained and implemented information into their didactic and clinical curricula, what priorities and resources shaped those decisions, and how the training occurred. Simultaneously, we gathered information about student training in fluoride treatment (a treatment applied in the dental office to reduce tooth decay) and tooth whitening (a popular esthetic treatment, also applied in the dental office) as comparisons to elicit varying approaches, motives, and priorities in making curricular decisions.

## Methods

We used a qualitative method to elicit underlying thought processes and narratives, as quantitative research on the subject was already available. We formulated the research as a series of case studies of dental hygiene education programs using telephone interviews of faculty (10). A trained master’s-level graduate student interviewed 32 participants from 19 dental hygiene training programs during 2008 and 2009. The University of Illinois at Chicago institutional review board approved this study. 

The dental hygiene programs were selected according to 2 characteristics: 1) length of program (2-year or 4-year) and 2) affiliation with a dental school (yes or no). From the 4 categories created, we randomly selected 12 programs from the eligible US dental hygiene programs listed on the website of the American Dental Hygienists’ Association (11) and invited program directors to be interviewed. When 6 programs in a category accepted an interview, we ceased invitations. Only 5 programs fit the 2-year affiliated category, so we invited all 5. The refusal rate in the 2-year unaffiliated category was high, so we stopped invitations after 4 accepted (Table 1). We recruited the program and clinical directors from each invited institution. Either person could designate a more qualified person to discuss the program’s tobacco use cessation activities. Five programs provided only 1 participant. Two participants in 1 program chose to be interviewed together. We recorded and transcribed the interviews and paid participants for their time. To maintain anonymity, we assigned a code to each school and participant and removed names from the transcripts.

**Table 1:** US Dental Hygiene Educator Program and Faculty Response Rates to Interview Request, by Length of Program and Affiliation With a Dental School, 2008–2009

Program Length/Affiliation With Dental School	No. of Eligible Programs	No. of Programs Invited	No. of Programs That Accepted	No. of Interviewees Who Accepted	No. of Interviewees Who Declined
2-Year/no	237	12	3	4	2
4-Year/no	30	13	6	11^a^	1
2-Year/yes	5	5	4	7	1
4-Year yes	24	7	6	11	1
**Total**	296^b^	37	19	33	5

^a^ Two people were interviewed together, for a total of 32 interviews.

^b^ Two hygiene programs were ineligible because of connections to the authors.

During the first interview, we asked participants about their programs’ strategies and processes to determine the content of their curriculum. We modified the interview protocol twice during data collection, adding questions about the clinical training of students and their evaluation and about the curricular priorities and objectives of the individual programs. We wanted to avoid responses calculated to show the program in a good light, so we avoided asking questions for which there was a “correct” response. The interviewer allowed participants flexibility to respond in their own words. 

To organize the data into case studies of programs, we extracted interview data individually and then collated and summarized the data into a program case (Table 2). The data analysis team consisted of 2 trained experts in dental education and a consulting organizational psychologist. The overall approach was standardized: the 2 analysts first examined data independently and then created a joint summary, discussing and resolving issues together. The consulting psychologist reviewed and critiqued each step and resolved disputes. In very few cases, participants provided conflicting information; it was usually clear which participant was more actively involved in the educational activity, and we took this version as our guide. We noted unresolved differences on the program summary sheet (Table 2). We rated conflicting data in the results as missing. Finally, we coded how and to what level each program evaluated student competence (Table 3).

**Table 2:** Steps in Qualitative Data Analysis of US Dental Hygiene Training Programs’ Approaches to Tobacco Dependence Treatment, 2008–2009

Activity	Performed by Whom/How Performed
Transcribed interviews	Master’s student.
Developed the interview summary form	The data analysts, the consultant, the master’s student, and 2 doctoral students read several transcripts to develop the interview summary form.
Compiled interview summary forms from each participant for each focus area^a^	Each data analyst independently filled out a form for each interview, using the transcripts. Then the members met to create 1 joint interview summary form for each transcript. This ensured that all relevant information was included. The consultant critiqued the form and the process.
Compiled program summary forms, which consolidated information from the interview summaries	Each data analyst independently filled out a form using the interview summaries. Then the members met to create 1 joint program summary form. The consultant critiqued the form and the process.
Coded each program for level of competence	The data analysts independently rated each program for the level of competence in each focus area. They then discussed disagreements and resolved them. They noted missing or unclear data.

^a^ Focus areas were presence of a “champion” who promoted the focus area in the program, didactic content taught, faculty training in the clinical application, clinical training of students, assessment of student clinical performance, which determined level of competence, faculty training in student clinical assessment, how information entered the system and was disseminated, how didactic and curricular decisions were made, difficulties with implementing, resources used or needed, description of program motives and priorities when making decisions.

**Table 3:** Dental Hygiene Programs’ (N = 19) Levels of Competence in Training Students in Tobacco Dependence Treatment, Fluoride Therapy, and Tooth Whitening, 2008–2009

Type of Training	Level 1: No Clinical Competence Determined, No. (%)	Level 2: Competence Determined Preclinically, No. (%)	Level 3: Competence Determined Clinically, No. (%)
Tobacco dependence treatment^a^	9 (47)	8 (42)	2 (11)
Fluoride therapy^b^	0	7 (37)	11 (58)
Tooth whitening	4 (21)	15 (79)	0

^a^ For tobacco dependence treatment, level 2 was defined as formal evaluation with a simulated patient or informal evaluation with a patient; level 3 was defined as formal competence evaluation with a patient.

^b^ One response missing.

## Results

Response rates of the programs to the interview request were lowest among 2-year programs unaffiliated with dental schools and highest among programs affiliated with dental schools. Response rates of the faculty were lowest among the 2-year unaffiliated programs (Table 1).

### Obtaining information and adoption of curriculum

All programs obtained information similarly (about tobacco dependence, tooth whitening, or fluoride) to make curricular decisions. Faculty learned of TDT through textbooks, journals, and professional meetings. A faculty member of 1 program mentioned state and university mandates to have a tobacco use cessation program. In many programs, individual faculty members had received continuing education in TDT and incorporated it into the program. Most respondents mentioned the American Dental Hygienists’ Association’s Ask, Advise, Refer initiative as a spur to curriculum development (12). The initiative modifies the 5As by emphasizing only 3 steps: 1) ask about tobacco use, 2) advise the patient to quit, and 3) refer the patient to a quit line for telephone counseling in support of cessation. There was limited mention of the 5As, although they are presented in the 3 foundational dental hygiene texts (13–15). Two dental hygiene programs were affiliated with dental schools that had received grants to implement TDT, and their faculty members mentioned the 5As.

Faculty members of all programs identified tobacco dependence, fluoride, and tooth whitening as topics appropriate for their curricula. Respondents indicated a standard process of bringing information to faculty meetings, where decisions were made to incorporate content into courses. The didactic component was delegated to the course director, and the clinical training component was delegated to the clinical supervisor.

### Implementation of clinical training

As faculty members of programs implemented curricula, they defined and structured the type of clinical training students would receive in each area. Generally, clinical training decisions were made by the clinical director or by the faculty member who was most interested in clinical training in the subject.

Dental hygiene training often includes preclinical or laboratory training for procedures before patient treatment, in which the student practices an activity on other students or in a simulation exercise. Fluoride therapy was universally taught first preclinically by having students apply topical fluoride treatments to one another before they treated patients. Tooth whitening also includes a preclinical or laboratory component; students were trained to make custom trays (plastic trays that fit closely around the teeth and hold fluid next to the teeth) in a laboratory setting. 

The equivalent of preclinical training in TDT was any exercise in which a student practiced providing TDT in a simulated setting, usually with another student or in a small group. Some programs provided case studies of TDT for students to discuss. One program had students write a script providing TDT to an imaginary patient. Eight programs provided this (preclinical) level of training in TDT. 

Definition of TDT varied by program. Many programs approached TDT as a form of patient education, providing the didactic content in a patient education or a special needs course for patients. Programs rarely mentioned referring smokers to the local tobacco quit line or providing patients with written material about quitting. Although respondents frequently mentioned the Ask, Advise, Refer model for TDT, they rarely mentioned the “refer” part as a training objective. They also never mentioned 5As in the context of chair-side tobacco dependence training, only as part of a didactic course.

Dentists were available to prescribe cessation medications in some programs. One program developed a tobacco use cessation kit, which included written material for the student and patient to assist with cessation. Hygiene students from 1 program affiliated with a dental school that provided a tobacco use cessation program were told to refer patients to this program and were offered the opportunity to learn and practice TDT in that clinic. Whether hygiene students in that program or in others were expected to provide patients with support to follow-up on the referral was unclear.

Preclinical training is followed by training with patients (clinical training). When discussing clinical training, participants almost always made reference to a structure used in dental hygiene education called ADPIE (assess, diagnose, plan, implement, evaluate) (16), the process of care for dental hygiene treatment that encourages student hygienists to attend to each part of the care process. Integration of TDT into clinics was typically structured through ADPIE.

Faculty members of most programs described integrating assessment of tobacco use into the oral health risk assessment (the first step of ADPIE). Asking about tobacco use was part of the patient health history. If a patient was identified as being a tobacco user, faculty members expected the student to address the tobacco use in the plan, and sometimes the computerized charts prompted that step. Faculty members of 17 of the 19 programs indicated that students were expected to educate tobacco users about the oral effects of tobacco use. The student was often referred to a “tobacco champion,” if available — someone who takes responsibility to ensure that tobacco dependence training is incorporated into the curriculum correctly — to assist with the dependence treatment. Students were often encouraged to link oral findings on the examination to tobacco use.

Programs varied on whether TDT was a required experience. A few participants noted that there were not enough tobacco users in the patient pool and, therefore, their programs did not require TDT for graduation. Some programs overcame the paucity of tobacco-using patients in creative ways. One program took students to a local veterans’ hospital; several programs allowed students to use a family member or friend who smoked instead of using a patient. Only 2 programs evaluated formal competence in tobacco use cessation.

Five programs identified tobacco champions who ran the didactic, preclinical, and clinical training of TDT. Students in those programs received more intense training in TDT than students from other programs. However, 2 programs reported the collapse or significant contraction of the TDT program when the attention of the champion was withdrawn. Programs with champions tended to rely on the champion to assess students’ clinical competence without training other faculty members.

The differences in training among fluoride training, tooth whitening training, and TDT were related to the counseling aspects of TDT and to the mission of dental hygiene. Providing fluoride therapy and tooth whitening are procedurally simpler than providing counseling. Furthermore, tooth whitening was not considered part of the dental hygiene mission and in many states was not part of the hygiene scope of practice, so when difficulties with clinical implementation were encountered, tooth whitening training was simply dropped. TDT was seen as part of the preventive mission of hygiene, but because it was not needed for most patients, it was deemed less important than training in fluoride treatment.

### Evaluation of student competence

We categorized programs into 3 levels on the basis of how they assessed student competence. Level 1 programs did not evaluate clinical competence. Level 2 programs either provided an informal evaluation (not required) or evaluated a simulated patient experience. Level 3 programs formally assessed competence as a student treated a patient and required this assessment for all students (Table 3).

Competence was evaluated at level 3 in TDT in only 2 programs, both of which had a tobacco champion. Almost half of the programs did not evaluate clinical competence in TDT. To achieve level 3, programs had to require students to provide TDT for a tobacco user and have a faculty member evaluate that provision; students accomplished this training by writing a case study describing how they helped a patient or family member with tobacco use cessation. Students first wrote a case assessment and then planned an intervention with the help of a supervisor. After providing the intervention, they wrote a detailed description of the intervention and how the patient received it and reviewed it with the supervisor, evaluating opportunities for improvement. They were then required to follow up with the patient 6 months later.

No programs required level 3 competence of their students for tooth whitening training. If clinical evaluation on a patient for fluoride treatment was not specifically mentioned, we coded the training as level 2, so the proportion of programs evaluating students to level 3 may have been higher than the 58%.

### Barriers to full clinical implementation of TDT

When asked what interfered with training students to full competence in TDT, faculty members reported lack of curriculum time, lack of tobacco users in their population, lack of trained faculty, lack of a dentist willing to prescribe medications, lack of interest by faculty or directors, and (rarely) faculty feeling it “wasn’t their place” to change patients’ smoking behavior. When asked what would encourage them to train students to full competence, faculty members indicated having a tobacco champion, having TDT be an accreditation requirement, and having examples to follow for competence evaluation.

### Faculty training

All programs reported some faculty training in fluoride treatment, tooth whitening, and tobacco dependence. However, since TDT is more complex than training in providing fluoride or tooth whitening treatments, the training often involved an individual faculty member obtaining formal training elsewhere and informing the rest of the faculty. Faculty members were not responsible for providing formal TDT training. Instead, they checked whether students asked about tobacco use and advised patients to avoid tobacco use, and assisted students in providing patient education about tobacco use.

## Discussion

We collected qualitative data about how dental hygiene programs implement TDT into their didactic and clinical curricula and the processes they use to make those decisions. The process used to implement and adapt curricular material was similar among programs, regardless of the type program or subject matter being implemented. Program directors were skilled at identifying information and integrating it into coursework. Programs taught clinical procedures first in a laboratory or simulated environment, and then students practiced the procedures on patients in the clinic under supervision, using the ADPIE structure as a guide. In the case of TDT, programs consistently required students to assess and record tobacco use and advise against it, although few programs formally evaluated those steps. Programs generally encouraged patient education about tobacco use and its oral effects. However, few programs taught students how to help a patient make a commitment to quit, or taught how to support a patient in quit strategies, as recommended by the USPHS (4). Only 2 programs ensured TDT competence in their graduates. This limited tobacco use intervention is consistent with the approach supported by the Ask, Advise, Refer model (12) and with quantitative research reports of dental hygiene education (17,18) and education among other health care professionals (19–24).

The degree to which health professionals should be educated in TDT is arguable. The participating programs approach minimally meeting the recommendations of Healthy People 2020 for tobacco use screening and cessation counseling in dental care settings (2) and the recommendations of the National Action Plan for Tobacco Cessation for “all clinicians . . . [to] have the knowledge, skills and support systems necessary to help their patients quit tobacco use” (3). It does not meet the recommendations of the USPHS that clinicians be prepared to both help patients become ready to quit and assist in the quitting process once they are ready (4). It also does not meet the recommendations of an international consensus paper advising that dental personnel be trained to provide intermediate care, which involves a 5- to 10-minute intervention consisting of brief motivational interviewing, building on readiness to quit, enlisting resources to support change, and including cessation medications (21). Although other barriers may inhibit dentists and hygienists from providing counseling as recommended, lack of training should not be a barrier.

If dental hygiene as a profession should decide to commit to graduating hygienists competent in TDT, this study has provided insight as to how this commitment could be implemented. Dental hygiene education can use ADPIE, an existing process, to facilitate the provision of full TDT. Counseling interventions could be structured to the ADPIE process, as oral hygiene instructions are. ADPIE could include assessing at each appointment a tobacco user’s attitude toward cessation, determining whether they need support to commit to a change or support to choose workable strategies, and providing one or the other. Tobacco use cessation is clearly related to the mission of dental hygiene to prevent disease, as expressed in the field’s accreditation standards (16). Providing models of training, TDT, and competence assessment would also facilitate this implementation.

Although a valuable resource in developing TDT competency is a tobacco champion, champions proved to be a mixed blessing. Training students to a higher level was associated with having a tobacco champion, but the tobacco use cessation programs tended to fail when the champion moved on because the tobacco programs were not fully integrated into the dental hygiene program with faculty buy-in and training. We recommend that all faculty be trained in the basics of helping a patient decide to quit and helping a patient choose quitting strategies.

This study has several limitations. The response rates from programs not affiliated with dental schools were low, especially those of 2-year programs. Faculty who considered themselves good at or interested in tobacco use cessation may have been overrepresented, which would have the effect of increasing our access to workable solutions. Allowing participants to tell about their experiences in their own words may have decreased the number of socially acceptable responses; however, this technique did not allow equivalent data to be collected from all participants, a common difficulty with multiple case analyses (10). Additionally, the interviews relied on participants’ memories or assumptions about processes, which may not have been accurate. Similarly, participants did not always fully respond to questions, resulting in some missing information. Finally, the focus of the interviews changed over time, so quantitative conclusions about prevalence of processes should be interpreted with caution. However, the processes among programs were so similar that we are confident that this study describes the range of implementations of tobacco use cessation in dental hygiene education.

Our data show how TDT has been altered and simplified from the public health guidelines (4), resulting in few programs training dental hygiene graduates to competence in TDT using all 5As. This information will help stakeholders understand which interventions may improve dental hygiene competence in TDT.
